# Development of Central Nervous System Autoimmunity Is Impaired in the Absence of Wiskott-Aldrich Syndrome Protein

**DOI:** 10.1371/journal.pone.0086942

**Published:** 2014-01-23

**Authors:** Marita Bosticardo, Silvia Musio, Elena Fontana, Stefano Angiari, Elena Draghici, Gabriela Constantin, Pietro L. Poliani, Rosetta Pedotti, Anna Villa

**Affiliations:** 1 TIGET, San Raffaele Scientific Institute, Milan, Italy; 2 Foundation IRCCS Neurological Institute “C.Besta”, Neuroimmunology and Neuromuscular Disorders Unit, Milan, Italy; 3 Department of Molecular and Translational Medicine, Pathology Unit, University of Brescia, Brescia, Italy; 4 Department of Pathology and Diagnosis, Section of General Pathology, University of Verona, Verona, Italy; 5 Milan Unit, Istituto di Ricerca Genetica e Biomedica, Consiglio Nazionale delle Ricerche, Milan, Italy; University of Münster, Germany

## Abstract

Wiskott-Aldrich Syndrome protein (WASP) is a key regulator of the actin cytoskeleton in hematopoietic cells. Defective expression of WASP leads to multiple abnormalities in different hematopoietic cells. Despite severe impairment of T cell function, WAS patients exhibit a high prevalence of autoimmune disorders. We attempted to induce EAE, an animal model of organ-specific autoimmunity affecting the CNS that mimics human MS, in *Was^−/−^* mice. We describe here that *Was^−/−^* mice are markedly resistant against EAE, showing lower incidence and milder score, reduced CNS inflammation and demyelination as compared to WT mice. Microglia was only poorly activated in *Was^−/−^* mice. Antigen-induced T-cell proliferation, Th-1 and -17 cytokine production and integrin-dependent adhesion were increased in *Was^−/−^* mice. However, adoptive transfer of MOG-activated T cells from *Was^−/−^* mice in WT mice failed to induce EAE. *Was^−/−^* mice were resistant against EAE also when induced by adoptive transfer of MOG-activated T cells from WT mice. *Was^+/−^* heterozygous mice developed an intermediate clinical phenotype between WT and *Was^−/−^* mice, and they displayed a mixed population of WASP-positive and -negative T cells in the periphery but not in their CNS parenchyma, where the large majority of inflammatory cells expressed WASP. In conclusion, in absence of WASP, T-cell responses against a CNS autoantigen are increased, but the ability of autoreactive T cells to induce CNS autoimmunity is impaired, most probably because of an inefficient T-cell transmigration into the CNS and defective CNS resident microglial function.

## Introduction

Wiskott-Aldrich syndrome (WAS) is a severe X-linked disorder characterized by microthrombocytopenia, eczema, immunodeficiency and increased risk of developing autoimmunity and lymphomas [1]. The protein encoded by the WAS gene, WASP, is a hematopoietic specific regulator of actin nucleation in response to signals arising at the cell membrane [2], [3]. In the last years several WASP mutations have been identified, resulting in a variety of clinical manifestations ranging from the relatively mild X-linked thrombocytopenia (XLT) to the classic full-blown WAS phenotype [4]–[6]. The hallmarks of classical WAS cases are represented by compromised humoral and adaptive immune responses. T-cell defects hamper both effector and helper functions because of the key role of WASP in T-cell activation and actin cytoskeleton remodeling upon TCR engagement [7]. As a consequence of impaired signaling through the TCR and co-stimulatory molecules, WASP-deficient T cells show defective proliferation and decreased secretion of IL-2 and Th-1 cytokines [7], [8]. These findings might help explaining the unbalanced Th1/Th2 cytokine production observed in patients and in the murine counterpart [7]. However, the precise relationship between T-cell abnormalities and autoimmunity in WAS patients remains to be fully elucidated. In fact in these patients, as in several other primary immunodeficient (PID) patients, immunodeficiency is often accompanied by the development of autoimmune disorders [9], reported to affect 25% to 72% of patients [10]–[12], irrespectively of WASP expression levels and disease severity [12].

We thus set out to analyze the role of WASP in a prototypical animal model of organ-specific autoimmunity, experimental autoimmune encephalomyelitis (EAE), an inflammatory demyelinating disease of the CNS mimicking multiple sclerosis (MS) [13]. Much evidence demonstrates that Th-1 and -17 cells activated against the myelin of the CNS drive the pathogenesis of both EAE and MS [14]. Furthermore, migration of autoreactive T cells through the blood-brain-barrier (BBB) into the CNS parenchyma and recruitment of inflammatory cells are crucial steps for the development and perpetuation of these diseases [15]. Because of its complexity, this model allowed us to explore the role of WASP both in the initiation phase of peripheral T-cell activation against self-antigens and in the consequent effector phase, in which autoreactive T cells migrate into the target organ. In the present paper we show that WASP-deficiency impairs the development of EAE by affecting both migration of autoreactive T cells in the CNS and activation of CNS resident cells, such as microglia.

## Materials and Methods

### Mice, peptides and EAE induction

WAS knock-out mice were generated as previously described [16]. In our study, we used 8–12 week old female BL6-wasnull (*Was^−/−^*) mice. Age/gender-matched C57BL/6N WT control mice were purchased from Charles River Laboratories Inc (Calco, Italy). *Was^+/−^* heterozygous female mice were generated by crossing *Was^−^* male mice and WT female mice and presented both WT and transgenic WAS alleles. EAE was induced as described [17] by subcutaneous immunization with MOG_35–55_ (100 µg/mouse) in Complete Freund's Adjuvant (CFA, Difco), containing 4 mg/ml of heat-killed Mycobacterium tuberculosis (Difco). All mice were injected i.v. with 200 ng/mouse of Bordetella Pertussis toxin (PTX, List Biological Lab) on day 0 and 2 post-immunization. Passive EAE was induced by i.v. injection of 5×10^6^ activated T-cell blasts, obtained from draining lymph nodes of MOG_35–55_-immunized mice 7–10 days after challenge and re-stimulated in vitro for three days with MOG_35–55_ (20 µg/ml) and IL-12 (1 ng/ml), and i.p. injection of 50 ng of PTX [18].

### Ethics statement

All mice were housed in specific pathogen free conditions and treated according to protocols approved by the Animal Care and Use Committee of the San Raffaele Scientific Institute (Institutional Animal Care and Use Committee protocols no. 318-406-557).

### Pathological studies

Mice were sacrificed 2 and 7 weeks after induction of EAE and brains and spinal cords were removed and fixed in 10% formalin. Four μm paraffin-embedded sections were used for routine Haematoxylin and Eosin (H&E) staining and sections were blindly analyzed (E.F., P.L.P.). Polyclonal/monoclonal specific antibodies for GFAP (Dako, Glostrup, Denmark), MBP (Chemicon, Temecula, CA), CD3 (Thermo-Scientific, Fremont, CA), Iba-1 (Wako, Osaka, Japan) and WASP (503 Ab, a kind gift from Profs H. D. Ochs, Seattle, WA, and L. D. Notarangelo, Boston, MA) have been applied to investigate reactive gliosis, demyelination, T-cell infiltration, presence of macrophages, microglial activation, and WASP expression. Immunostains were revealed by Real EnVision Rabbit HRP Labelled Polymer system (Dako) and Rat on Mouse HRP polymer kit (Biocare medical) using 3,3′-diaminobenzidine tetrahydrocloride (DAB, 0.05%) as a chromogen and Haematoxylin as counterstaining. Double immunostains were performed as previously described [19]. Chromogen reaction was developed by Ferangi Blue Chromogen Kits (Biocare Medical, Concord, CA) and diaminobenzidine. Nuclei were counterstained with methyl green. The degree of inflammation has been expressed as ratio between distribution of the inflammatory cells within CNS parenchyma (measured as total area occupied by inflammatory cells) and the mean area of the spinal cord, obtained analyzing the total area of 8 different spinal cord sections at different levels. Demyelination was evaluated by measuring the percentage of demyelinated areas over the total area of white matter for each single spinal cord section. An average of 12 spinal cord sections for each mouse were studied. Digital images and analysis were obtained by Olympus DP70 camera mounted on an Olympus Bx60 microscope, using CellF Imaging software (Soft Imaging System GmbH).

### Antigen-specific T-cell proliferation assay

Draining lymph node cells (LNCs) were isolated from *Was^−/−^* and WT mice and cultured *in vitro* with MOG_35–55_ (0.1–100 µg/ml), Concanavalin A (ConA; 4 µg/ml) or medium alone. Cells were cultured at a density of 5×10^5^ cells/well in 200 µl RPMI 1640 (EuroClone) supplemented with 10% FBS, L-glutamine (2 mM), sodium pyruvate (1 mM), nonessential amino acids (0.1 mM), penicillin (100 U/ml), streptomycin (100 U/ml), 2-ME (5×10^−5^ M). After 72 h of incubation at 37°C with 5% CO_2_, cultures were pulsed for 18 h with 0.5 µCi/well of [^3^H] thymidine before harvesting.

### Cytokine analysis

Splenocytes were harvested from *Was^−/−^* and WT mice and cultured in the same conditions as described above at a concentration of 3.5×10^6^ cells/well. Supernatants from *in vitro* cultured splenocytes were collected after 48 and 96 h and analyzed by multiplex assay (Bioplex, Bio-rad, Hercules, CA) according to the manufacturer's instructions. Serum cytokine concentrations were measured by Bioplex reader (Bio-Rad) on sera diluted 1∶4.

### Measurement of serum antibody responses

Blood was collected from the tail vein of *Was^−/−^* and WT mice before and 2–7 weeks after the induction of EAE. MOG-specific IgG, IgG1, IgG2a, IgG2b and IgG3 antibodies and total IgE antibodies were measured on serum samples by ELISA assay as described [17]. Briefly, 96-well plates (Immunol, Thermo Labsystems, Franklin, MA) were coated overnight at 4°C with 0.1 ml of MOG_35–55_ diluted in 0.1 M NaHCO3 buffer pH 9.5 at a concentration of 0.01 mg/ml. The plates were blocked with PBS 10% FBS (blocking buffer) for 2 h. Samples were diluted in blocking buffer at 1∶100 and antibody binding was tested by the addition of peroxidase-conjugated monoclonal goat anti-mouse IgG, IgG1, IgG2a, IgG2b and IgG3 (Southern Biotechnology Associates, Birmingham, AL), each at 1∶5000 dilution in blocking buffer. Enzyme substrate was added and plates were read at 450 nm on a microplate reader.

### Flow cytometry analysis

Activated T cells from MOG-immunized WT or *Was^−/−^* mice were re-stimulated *in vitro* for 3 days with MOG_35–55_ peptide in the presence of antigen presenting cells (irradiated splenocytes). Dead cells were then eliminated by Ficoll-Paque (GE Healthcare) gradient. T-cell blasts so obtained were labeled with monoclonal antibodies for α_4_-integrin (PS/2 clone, kindly provided by Dr. Eugene Butcher, Stanford University), Lymphocyte Function-associated Antigen (LFA)-1 (anti-α_L_-chain; clone TIB213 from American Type Culture Collection/ATCC, VA, USA), P-Selectin-Glycoprotein Ligand (PSGL)-1 (clone 4RA10, kindly provided by Dr. Dietmar Vestweber, Max Plank Institute, Germany), L-selectin (Mel-14 clone, ATCC) and CD44 (IM/7 clone, ATCC). Isotype-matched antibodies were used as controls. Antibodies were detected with secondary goat anti-rat PE antibody (Biolegend). At least 10,000 events were collected by flow cytometry (FACScalibur, Becton Dickinson) using the Cell Quest software and analyzed with the FlowJo software (Tree Star Inc.).

### Adhesion assay

Recombinant murine Intercellular Cell Adhesion Molecule (ICAM)-1 (1 µg/ml, R&D Systems) was coated over night at 4°C on 18-well glass slides. WT or *Was^−/−^* T-cell blasts were collected and re-suspended 4×10^6^/ml in adhesion buffer (PBS + Ca^++^/Mg^++^ 1 mM + 10% FBS, pH 7.2). Twenty μl of cell suspension were added to each well and cells were allowed to spontaneously adhere on ICAM-1 for 10 min at 37°C. In some wells, CXCL12 chemokine (R&D Systems) was added at the final concentration of 0.5 µM, and rapidly-induced adhesion was measured after 5 min [20]. Slides were then immediately washed and fixed in ice-cold PBS 1.5% glutaraldehyde and computer-assisted enumeration of bound cells in 0.2 mm^2^ was performed.

### ImageStream data acquisition and analysis

WT or *Was^−/−^* T-cell blasts were stimulated with CXCL12 chemokine or control buffer for 5 min at 37°C. Two and a half million cells were then incubated with 10 µg/ml anti-LFA-1 antibody for 30 min on ice. After washing, cells were stained with PE-conjugated secondary antibody. Stained cells were re-suspended in PBS for the ImageStream analysis. Images were acquired on the ImageStream imaging cytometer System 100 (Amnis Corporation, Seattle, WA, USA). Images of fixed cells were collected and analyzed using ImageStream data exploration and analysis software, and LFA-1 clustering was evaluated as previously described [21]. Briefly, cells were divided in uniform (uniform distribution of fluorescence), clustered (small spots of fluorescence), and caps (big clusters of fluorescence) cells in accordance with LFA-1 distribution pattern.

### Intravital microscopy in brain pial vessels

Intravital microscopy experiments were performed as described [22], [23]. Briefly, WT mice were injected intraperitoneally with 12 µg lipopolysaccharide (LPS) 5–6 h before starting the intravital experiment. Animals were then anesthetized and a heparinized PE-10 catheter was inserted into the right common carotid artery toward the brain. In order to exclude non-cerebral vessels from the analysis, the right external carotid artery and pterygopalatine artery were ligated. The scalp was reflected and a 24 mm×24 mm coverslip was applied and fixed with silicon grease. A round camera with 11 mm internal diameter was attached to the coverslip and filled with water as previously described [22]. The preparation was placed on an Olympus BX50WI microscope and a water immersion objective with long focal distance (focal distance 3.3 mm, NA 0.5∞) was used. Blood vessels were visualized through the bone by using fluorescent dextran. Two-three million WT or *Was^−/−^* T-cell blasts were labelled for 2 min at 37°C with red (5-(and-6)-(((4-chloromethyl)benzoyl)amino) tetramethylrhodamine (CMTMR), re-suspended in PBS and slowly injected into the carotid artery by a digital pump. Images were visualized and recorded as for mesenteric vessels. Vessel diameter (D), haemodynamic parameters and velocities of rolling were determined by using a PC-based system as previously described [22], [23]. Lymphocytes that remained stationary on venular wall for ≥30 s were considered adherent. At least 100 consecutive cells/venule were examined. Rolling and firm arrest fractions were determined as the percentage of cells that rolled or firmly arrested within a given venule on the total number of cells entering the venule.

### Transwell migration assay

To assess LN cell migration in response to CXCL12 (Peprotech, Rocky Hill, NJ), total LN cells were isolated from 15 WT and 15 *Was^−/−^* mice 10 days after MOG_35–55_-immunization and seeded in the upper well of a 5 µm transwell (Corning Costar, Corning, NY) at the concentration of 5×10^6^/ml in 100 µl of medium. In the bottom well, we placed 600 µl of complete medium supplemented with 250 ng/ml of CXCL12. Cells were incubated for 3 h at 37°C, and cells migrated to the lower well were counted. Migration activity was calculated as percentage of migrated cells over the input cell number.

### Statistical analysis

Unless otherwise stated, Student's t-test, two tailed, was used to compare the results between two groups. In all tests, *P*<0.05 was considered statistically significant.

## Results

### *Was^−/−^* mice are resistant against EAE

**Figure 1:**
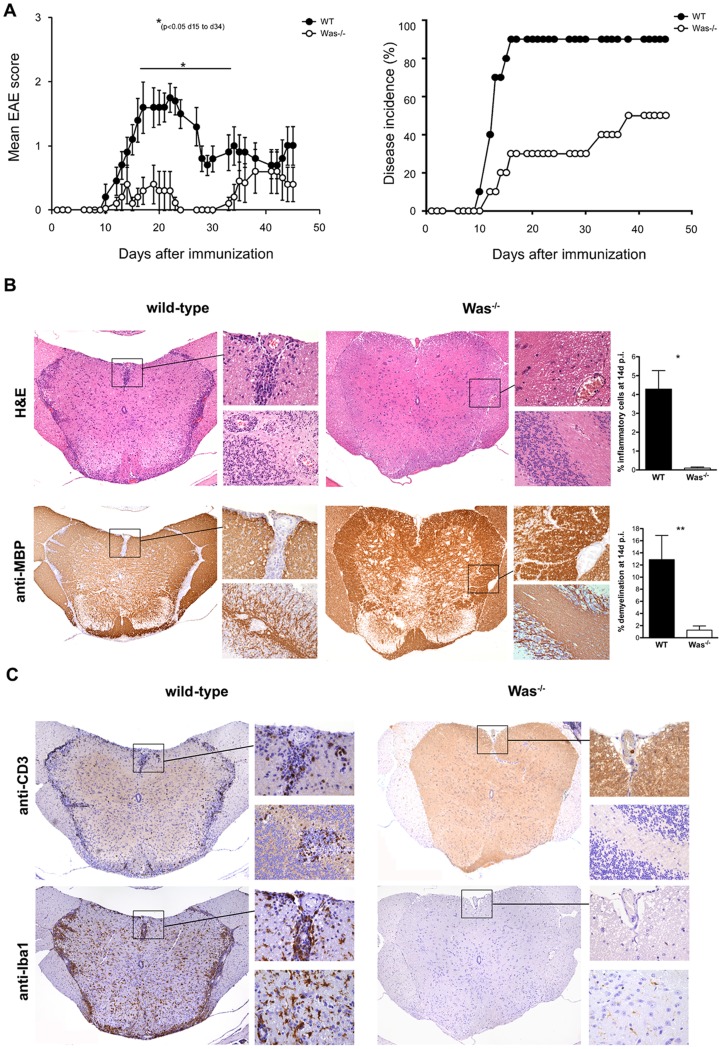
*Was* ^−^^*/*−^ mice are resistant against EAE. **A**, EAE was induced in *Was* ^−*/*−^ (*white circles*) and WT mice (*black circles*) with MOG_35–55_ peptide. Data represent mean clinical scores of 10 mice/group ± SEM. The experiment is representative of two independent experiments. Statistical analysis was performed with Sum Rank Test. ^*^ *P*<0.05. **B**, Histopathological analyses were performed on spinal cords and brains obtained from *Was* ^−*/*−^ and WT mice two weeks after the induction of EAE. Representative 5–8 mice per group were analyzed. H&E staining (*upper panels*) and anti-MBP immunostain (*lower panels*) highlight inflammatory changes and myelin damage, respectively. Insets report in detail inflammatory foci and demyelinated areas within spinal cords (*upper insets*) and brain parenchyma (*lower insets*), respectively. Graphs show percentage of inflammatory cell infiltration and demyelination in spinal cords of WT and *Was* ^−*/*−^ mice. ^*^ *P*<0.05; ^**^ *P*<0.01. **C**, CD3 (*upper panels*) and Iba-1 (*lower panels*) immunostains highlight T-cell infiltration and both macrophages and microglial activation, respectively. Details of the staining as for the previous panels are reported in the insets. In all panels serial sections of representative CNS sample are reported. Panels are 4× and insets 20× original magnification.

To study the development of autoimmune CNS demyelination in WASP deficiency, we induced chronic EAE with MOG_35–55_ peptide in *Was* ^−/−^ and WT control mice. In two consecutive experiments, we observed significant resistance against EAE in *Was* ^−/−^ mice (Figure 1A and Table 1). The disease incidence was reduced, the onset of EAE was delayed and the clinical severity was significantly reduced in *Was* ^−/−^ mice as compared to WT mice. In line with these clinical findings, neuropathological analysis of brain and spinal cord obtained from mice during the acute phase of EAE (14 days p.i.) showed a marked reduction of both CNS inflammation and demyelination in *Was* ^−/−^ as compared to WT mice (Figure 1B). In particular, the percentage of inflamed tissue within the CNS parenchyma and the percentage of demyelinated white matter area in the spinal cord of *Was* ^−/−^ mice were significantly reduced (*P*<0.05 and *P*<0.01, respectively) compared to those of WT mice (Figure 1C). Of note, immunostains for CD3 and Iba-1 revealed a dramatic decrease of T cells infiltrating the CNS parenchyma (Figure 1C, upper panels) in *Was* ^−*/*−^ mice as compared to WT mice, and microglial cells, which in WT mice with EAE were intensely activated and expressed WASP (Figure S1), were only weakly or not activated in the CNS of *Was* ^−*/*−^ mice (Figure 1C, lower panels). The differences in the extent of CNS inflammation and demyelination between *Was* ^−*/*−^ and WT mice observed 14 days after EAE induction were no longer significant at a later time point of EAE (48 days p.i.) (data not shown), when WT mice were in recovery and the differences in EAE severity between *Was* ^−*/*−^ and WT became less evident (Figure 1A and Table 1).

**Table 1:** Clinical traits of EAE in *Was* ^−*/*−^ and wild type mice.

Mice	Incidence (%)	Onset (day)	Disease score at peak^a)^	Maximum disease severity	Cumulative disease score
**Exp 1**					
**WT**	10/10 (100)	13±0.6	1.7±0.2	2.3±0.3	28.4±4.7
***Was*** ^**−**^^***/*−**^	5/10 (50)	22.6±3.9	0.3±0.3^***^	1.2±0.4^*^	7±2.8^***^
**Exp 2**					
**WT**	10/10 (100)	9.8±0.6	3.4±0.3	3.5±0.3	47±6.3
***Was*** ^**−**^^***/*−**^	6/10 (60)	18.5±4.6^*^	0.9±0.5^***^	1.7±0.6^**^	18.4±8.3^*^

Data are shown as mean ± SEM. ^a)^Mice reached a peak of disease severity at day 22 after immunization in Experiment 1 and at day 14 in Experiment 2. ^*^ *P*<0.05, ^**^ *P*<0.01, ^***^ *P*<0.005 *vs Was* ^−*/*−^ group.

### *Was* ^−*/*−^ mice develop a normal peripheral response against myelin antigen

**Figure 2:**
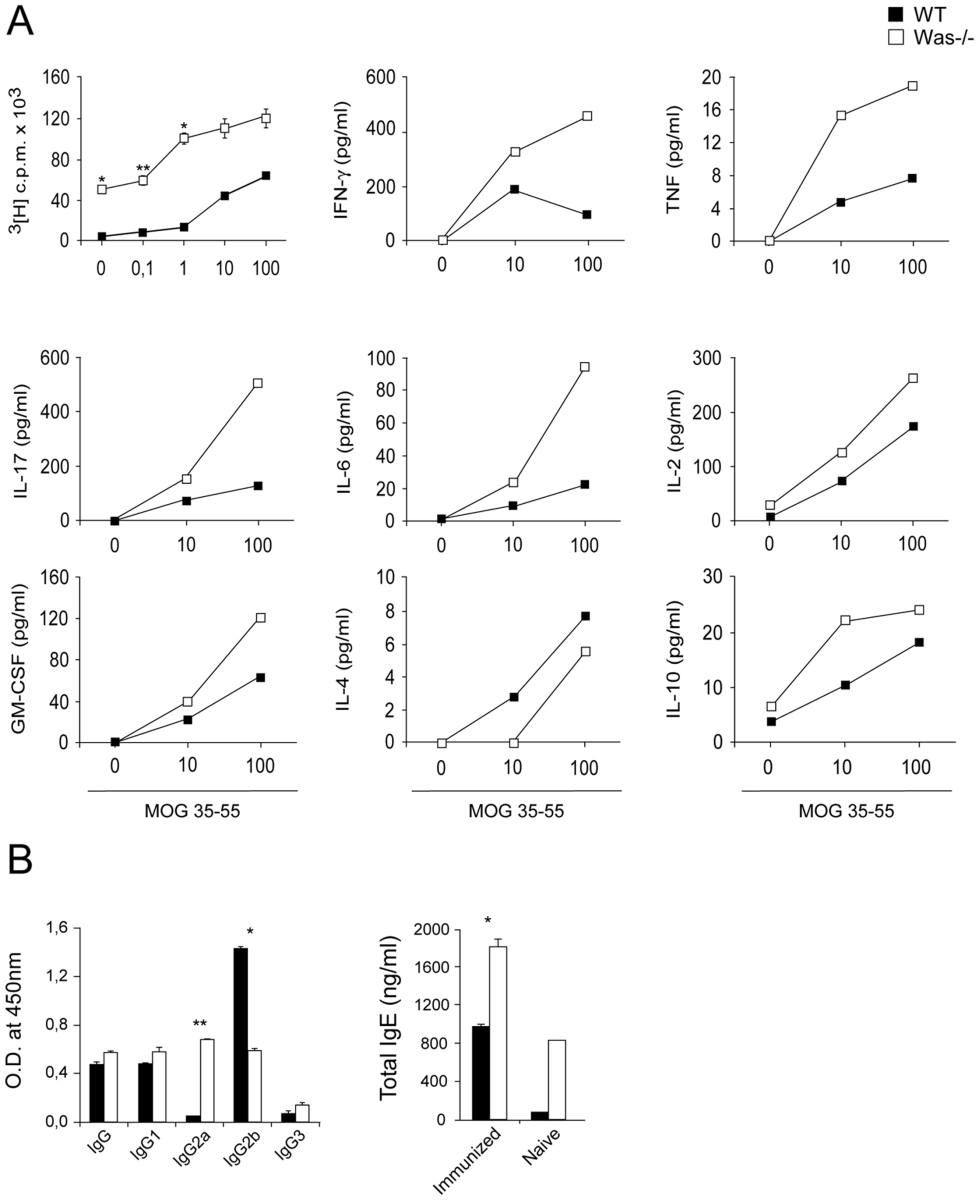
Peripheral T-cell and antibody response against myelin antigen in *Was* ^−^^*/*−^ mice. **A**, LNCs and splenocytes were harvested from *Was* ^−*/*−^ and WT mice (5 mice/group) two weeks after EAE induction and pooled cells were cultured *in vitro* with increasing doses of MOG_35–55_ peptide or medium alone. [^3^H] thymidine was added to LNCs cultures 72 h after stimulation (*upper left corner*). Cytokine levels were measured on supernatants from splenocytes 48 h after stimulation. **B**, Antigen-specific IgG antibodies (*left panel*) and total IgE antibodies (*right panel*) were measured in duplicate in sera obtained from mice (10 mice/group) 6 weeks after EAE induction. Data are representative of one of the two independent experiments that gave similar results. ^*^ *P*<0.05; ^**^ *P*<0.01.

Given the marked resistance against EAE displayed by *Was* ^−*/*−^ mice, we analyzed the peripheral immune response against myelin antigen in WASP deficiency. We first examined the ability of T cells from *Was* ^−*/*−^ mice to respond against MOG_35–55_. Antigen-induced T-cell proliferation in LNCs from mice immunized for EAE was higher in *Was* ^−*/*−^ mice than in WT mice (Figure 2A). Furthermore, antigen-stimulated spleen cells from *Was* ^−*/*−^ mice produced more Th-1 and Th-17 cytokines, such as IFN-γ, IL-17, TNF-α and IL-6, which are known to play a key role in the pathogenesis of EAE [24] (Figure 2A). GM-CSF, recently shown to play an essential function in the initiation of autoimmune neuroinflammation in EAE [25], and IL-2 were also increased in splenocytes of *Was* ^−*/*−^ mice upon antigen stimulation as compared to control mice. Of note, IL-10, known to play suppressive functions, was also found increased in mutant mice.

The analysis of the IgG antibody response against MOG_35–55_ revealed that there were not significant differences in serum antigen-specific IgG antibody titers among *Was^−/−^* and WT mice. However, the analysis of IgG antibody isotypes showed a decrease of antigen-specific IgG2b and an increase of IgG2a antibody titers in the serum of *Was^−/−^* mice as compared to those of WT mice (Figure 2B). The increased antibody switch towards the IgG2a isotype and the decrease in IgG2b subclass observed in *Was^−/−^* mice could be related to the higher levels of IFN-γ produced by activated splenocytes of *Was^−/−^* mice, as this cytokine is known to promote antibody class switch towards IgG2a while inhibiting IgG2b production [26]. Additionally, total IgE antibody concentrations were increased in the serum of both immunized and non-immunized *Was^−/−^* mice as compared to WT mice.

### Defects in both autoreactive T cells and host compartments contribute to the resistance against EAE of *Was^−/−^* mice

**Figure 3:**
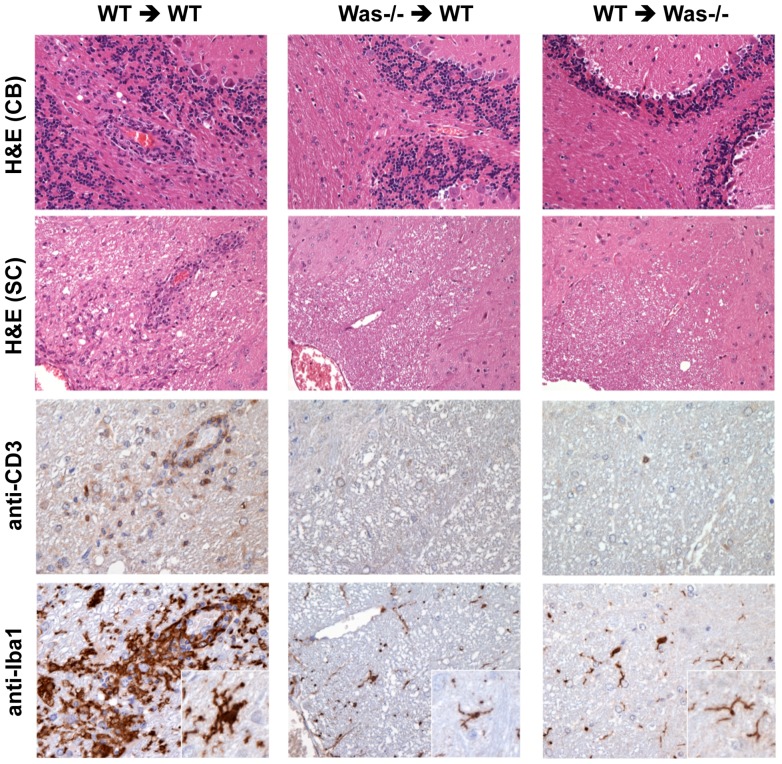
Histological features of brain and spinal cord sections from passively induced EAE (WT in WT, WT in *Was^−/−^* and *Was^−/−^* in WT). Samples were obtained 3 weeks after transfer and stained with H&E (*upper panels*; CB, cerebellum and SC, spinal cords), anti-CD3 and anti-Iba1 (*lower panels*). Insets show in detail representative microglial cells within spinal cords. Upper panels are from 10×, lower panels from 20× and insets from 40× original magnification.

As we did not observe defects in priming of autoreactive T cells in *Was^−/−^* mice, we wanted to ascertain the ability of these cells in inducing disease when transferred in non-immunized recipient mice. Adoptive transfer of MOG_35–55_-activated T cells from WT mice induced EAE in the majority of WT recipient mice (3/5, 60%), and neuropathological analysis of spinal cord and brains obtained 3 weeks after adoptive transfer showed infiltrating T cells and macrophages in the CNS parenchyma, along with microglia activation (Table 2 and Figure 3, left panels). Conversely, T cells from *Was^−/−^* mice failed to induce EAE in WT recipient mice (0/4, Table 2). Neuropathological analysis of spinal cords and brains of these treated mice confirmed the clinical results, as we could observe complete absence of infiltrating T cells and macrophages, along with absence of microglia activation (Figure 3, middle panels). These results demonstrate that, despite a normal and even increased generation of Th-1 and Th-17 response against MOG_35–55_ in *Was^−/−^* mice, autoreactive T cells from these mice were not able to induce EAE even when adoptively transferred in WT recipient mice. Surprisingly, adoptive transfer of myelin autoreactive T cells from WT mice failed to induce disease in *Was^−/−^* mice (0/5, Table 2 and Figure 3, right panels), and, accordingly, infiltrating T cells were absent in the CNS of these treated mice and microglia was scarcely/not activated.

**Table 2:** Passive transfer of EAE.

Donor	Recipient	Number of animals	Incidence (%)	Day of onset	Maximum disease severity
WT	WT	5	3/5 (60)	14±0^a)^	1.3±0.3^a)^
WT	*Was^−/−^*	5	0/5 (0)	-	0
*Was^−/−^*	WT	4	0/4 (0)	-	0

EAE was induced with injection of 5×10^6^ T-cell blasts per mouse derived from WT or *Was^−/−^* mice actively immunized with MOG_35–55_ peptide. ^a)^Data represent mean ± SEM for those mice that developed the disease.

**Figure 4:**
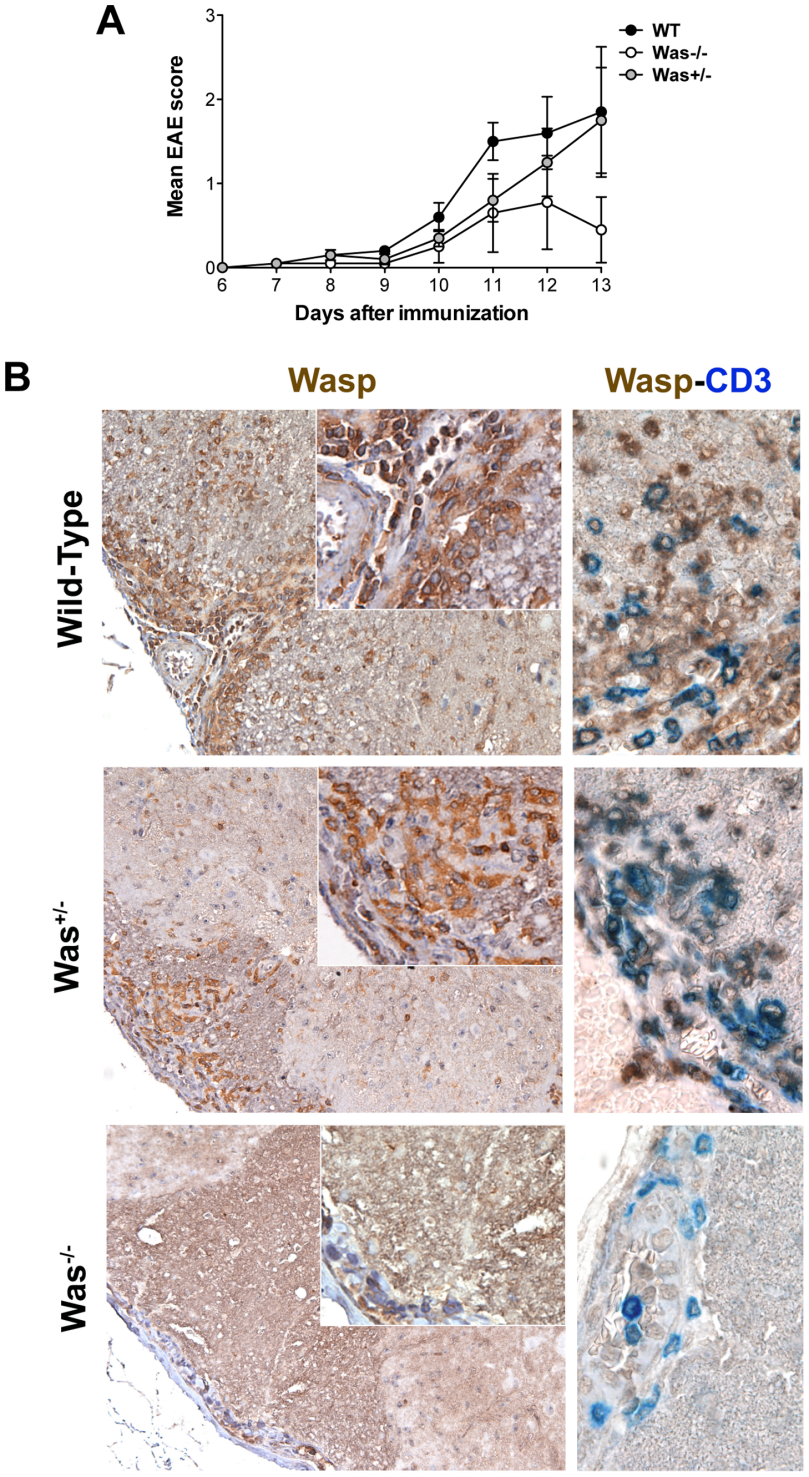
*Was^+/−^* heterozygous mice are not resistant to EAE induction. **A,** EAE was induced in *Was^−/−^* (*white circles*), *Was^+/−^* (*grey circles*) and WT mice (*black circles*) with MOG_35–55_ peptide. Data represent mean clinical scores of 5 mice/group ± SEM. **B,** Histopathological analyses were performed on spinal cords obtained from *Was^−/−^*, *Was^+/−^* and WT mice 14 days after the induction of EAE. WASP (*left panels*) and WASP-CD3 (*right panels*) immunostains highlight WASP expression in total CNS infiltrates and in T-cell infiltrates, respectively. Details of the staining are reported in the insets. In all panels serial sections of representative CNS samples are reported. Panels for WASP single stainings are 20×, insets 40× and WASP-CD3 double stainings 60× original magnification.

On the other hand, absence of disease transfer in WT recipient mice injected with *Was^−/−^* T cells suggests a defective migration of *Was^−/−^* T cells in the CNS of recipient mice. To verify this hypothesis, we induced EAE in *Was^+/−^* heterozygous mice, in which WASP-positive and -negative cells coexist, and evaluated their clinical scores and WASP expression in T cells from peripheral lymphoid organs and CNS (Figure 4). In parallel, WT and *Was^−/−^* mice were immunized as controls. As we reported in our previous experiments, *Was^−/−^* mice were resistant to EAE and showed only mild or no clinical score, while WT animals started showing signs of the disease at day 10 post-immunization and were all affected by day 13 (Figure 4A). Conversely, *Was^+/−^* heterozygous mice showed a clinical phenotype intermediate between WT and *Was^−/−^* mice (Figure 4A). In these mice a mixed population of WASP-positive and -negative T cells persisted in the periphery before and after MOG-immunization (Figure S1). Neuropathological analysis of spinal cords from *Was^+/−^* heterozygous mice conducted 14 days after EAE induction revealed that inflammatory infiltrates expressed WASP. Conversely, in *Was^−/−^* mice immunized for EAE, in which we did not detect inflammatory infiltrates within the CNS parenchyma, as expected there was no expression of WASP, and the few CD3-positive cells found in their spinal cords were localized in the vessels or in the leptomeningeal space (Figure 4B, bottom row). These results strongly suggest that WASP is required for the proper migration of immune cells from peripheral lymphoid tissues into the CNS (Figure 4B, middle row).

Taken together, these results suggest that deficiency of WASP affecting both the migration of autoreactive T cells into the CNS parenchyma and the activation of host compartment cells, such as microglia, could be responsible for the resistance against EAE observed in *Was^−/−^* mice.

### *Was^−/−^* T-cell blasts had no reduction of integrin-dependent adhesion *in vitro* and *in vivo*

**Figure 5:**
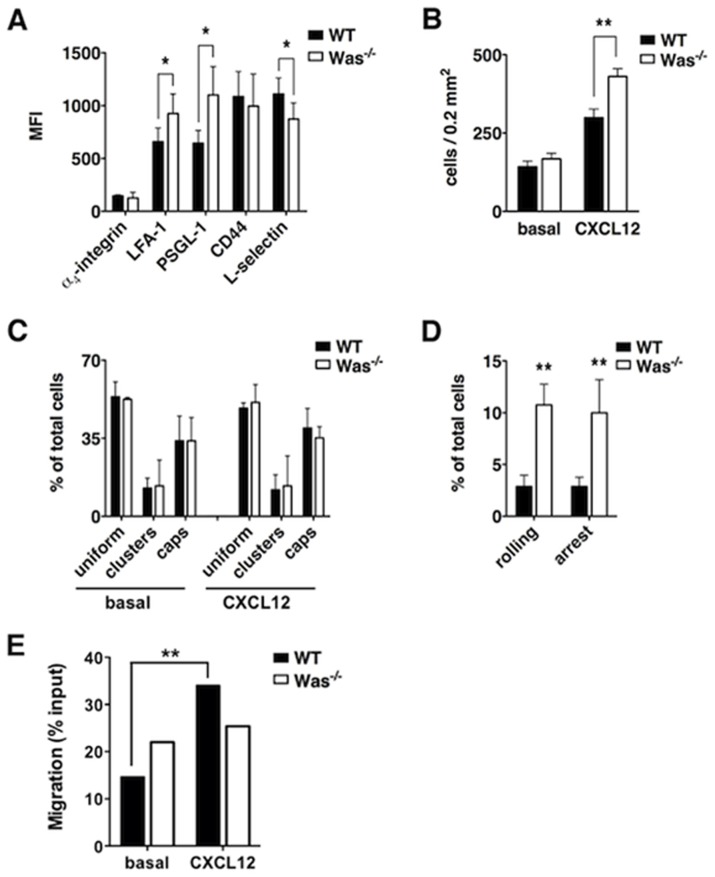
Adhesive properties of MOG_35–55_-primed WT and *Was^−/−^* T-cell blasts. **A,** The expression of LFA-1, α_4_-integrin, PSGL-1, CD44 and L-selectin was analyzed by flow cytometry on MOG_35–55_-primed T-cell blasts obtained form WT or *Was^−/−^* immunized mice. The mean fluorescence intensity (MFI) of staining is shown. **B,** WT or *Was^−/−^* MOG_35–55_-primed T-cell blasts were left to adhere spontaneously on slides coated with purified ICAM-1, with or without CXCL12. **C,** WT or *Was^−/−^* MOG_35*–*55_-primed T-cell blasts were stimulated with CXCL12 chemokine or control buffer and then labeled with anti-LFA-1 antibody and LFA-1 distribution on cell surface was analyzed with the ImageStream system. **D,** WT or *Was^−/−^* MOG_35–55_-primed T-cell blasts were injected in the right carotid of LPS-treated mice to analyze their interaction with brain pial vessels expressing ICAM-1, VCAM-1 and endothelial selectins [22]. Rolling interactions and stable adhesions were evaluated by analyzing at least 100 cells/venule. In all panels, data are mean ± SD of three independent experiments. **E,** WT or *Was^−/−^* LN cells isolated from MOG_35–55_-immunized mice were seeded in transwells and induced to migrate in the presence or absence of CXCL12 for 3 h. Frequency of cells migrated to the bottom wells of transwell plates are indicated in the graph. Each bar is representative of a pool of 15 mice. Statistical analysis was performed with Fisher's Exact Test. ^**^ *P*<0.01.

Because adoptive transfer of autoreactive T cells from *Was^−/−^* mice in WT recipient mice failed to induce EAE (Table 2 and Figure 3), we sought investigating whether the adhesive capacities of *Was^−/−^* T cells were compromised. We first performed flow cytometric analysis and found that T cells from *Was^−/−^* mice express significantly higher levels of integrin LFA-1 and mucin PSGL-1, but had lower L-selectin expression (Figure 5A). These results suggested that *Was^−/−^* T-cell blasts have a more activated phenotype and presumably higher adhesion capacity in inflamed vessels when compared to WT cells. No differences in the expression of α_4_-integrin and CD44 were found between the two populations (Figure 5A). Because LFA-1 integrin has been previously shown to mediate T-cell adhesion in inflamed brain vessels [22], we next analyzed the ability of T-cell blasts from WT and *Was^−/−^* mice to adhere *in vitro* on purified ICAM-1 after CXCL12-mediated triggering of adhesion. Our results showed that *Was^−/−^* T cells display a significantly higher adhesion on purified ICAM-1 when compared to WT cells (Figure 5B), suggesting that LFA-1 integrin is already activated in *Was^−/−^* T cells. Interestingly, the increased adhesiveness was not associated to an increase in LFA-1 clustering on *Was^−/−^* T-cell surface, as evaluated with ImageStream analysis (Figure 5C), suggesting that activated *Was^−/−^* T cells may have an increase in LFA-1 affinity compared to WT cells.

We next studied the adhesion capacity of *Was^−/−^* T cells *in vivo* by performing intravital microscopy in inflamed pial venules in an experimental model mimicking vascular inflammation occurring during early phases of EAE [22]. In this model, pial vessels express adhesion molecules such as ICAM-1, vascular cell adhesion molecule (VCAM)-1, E-selectin and P-selectin, as previously described [22], [27]. In line with the data obtained in *in vitro* adhesion assays as well as in flow cytometry experiments, we found that *Was^−/−^* T cells had an increased ability to roll and adhere in inflamed pial vessels when compared to WT cells (Figure 5D). In order to test whether increased adhesion capacity of *Was^−/−^* T cells corresponds to an augmented migratory ability of these cells, we performed a transwell migration assay in response to CXCL12 in WT and *Was^−/−^* LN cells isolated from MOG_35–55_-immunized mice (Figure 5E). Our results show that even if spontaneous migration was increased in *Was^−/−^* LN cells, the specific migration to CXCL12 was dramatically reduced in *Was^−/−^* LN cells, as compared to WT cells. Taken together, these results show that *Was^−/−^* T cells have increased adhesion capacity compared with WT T cells but reduced ability to transmigrate in response to chemokine signals.

## Discussion

The data reported in this study provide evidence that WASP is required for the development of EAE. In the absence of WASP, mice were markedly resistant against EAE. Resistance against EAE was present despite the increased autoreactive Th-1 and Th-17 cell responses against the self myelin antigen MOG_35–55_ developed by *Was^−/−^* mice.

WASP-deficient mice develop overt signs of autoimmunity only later in life in some specific background [28], even though most of these mice exhibit elevated titers of circulating autoantibodies in their sera [19], [28]–[30]. In our study, we used an induced model of organ-specific T cell-mediated autoimmunity to analyze the role of WASP in the development of autoimmunity. Several groups reported that WASP-deficient T cells fail to undergo a normal polarization, and that cytokine secretion from these cells is impaired [31]–[33]. This could either result from an indirect effect of WASP on gene transcription factors associated with cytokine production, or indicate an important post-transcriptional role for WASP in T-cell effector functions [8]. Moreover, *in vivo* evidence revealed that immune cells of WASP-deficient mice have motility defects, as DC homing from the periphery is kinetically and quantitatively impaired [34], [35]. *Was^−/−^* mice also display impaired DC priming of T cells due to defects in cell-to-cell interactions [34], [36]. In our model, LNCs from *Was^−/−^* mice proliferated more than those from wild type mice when stimulated with the self myelin antigen, and splenocytes produced more IFN-γ, IL-17, IL-6 and GM-CSF, cytokines that are all known to have a crucial role in the development of EAE. Of note IL-10, which is known to play a suppressive function in several autoimmune disorders including EAE, was also increased in *Was^−/−^* mice. The mechanisms underlying the increased immune responses against self MOG still need to be elucidated. However, it is possible that the reduced function and homeostasis of naturally occurring regulatory T cells (Tregs), which has been previously reported in *Was^−/−^* mice and WAS patients [29], [37]–[39], could be associated with these findings. However, even in the presence of an increased self antigen response in *Was^−/−^* mice, they were still resistant to develop EAE, indicating that impaired effector functions of *Was^−/−^* T cells are likely responsible of the absence of disease in the CNS of *Was^−/−^* mice.

In fact, although myelin-autoreactive *Was^−/−^* T cells exhibited an increased activation, the absence of WASP hampered the ability of these cells to induce CNS disease, also after passive transfer into recipient WT mice. Both the ability of T cells to traffic through the inflamed BBB into the CNS parenchyma and to migrate to and from secondary lymphoid organs, such as lymph nodes, might be impaired in WASP deficient mice, as proven by the fact that *Was^+/−^* heterozygous mice are not resistant to EAE induction and present only WASP-positive infiltrates in the CNS parenchyma. These defects appear not be associated with a decreased expression of adhesion molecules in *Was^−/−^* T cells. Indeed, we did not observe in *Was^−/−^* T cells defective expression of molecules such as LFA-1, integrin α_4_β_7_ and CD44, in accordance with data already published [40]. Nevertheless, we observed a dramatic decrease of infiltrating T cells and macrophages in the CNS of *Was^−/−^* mice, both in active induced EAE and adoptive transfer EAE. These data point to an inefficient recruitment of cells and macrophages in the CNS of *Was^−/−^* mice. However, WASP-deficient T cells display an increased chemokine-induced adhesion on ICAM-1 and a more efficient sticking on inflamed endothelium in brain microcirculation when compared to WT blasts. These data are in agreement with recent results showing that WASP-deficient T cells display an increased adhesion on ICAM-1 *in vitro* [41]. The increased adhesion of *Was^−/−^* T cells is in agreement with our recent data showing that CDC42, which is an upstream regulator of WASP, is involved in the negative control of integrin activation and T-cell adhesion *in vitro* and *in vivo* [20]. Thus, the lack of WASP inhibits CDC42 function leading to an increase of integrin activation and T-cell adhesion. Despite their increased capacity to adhere *in vitro* and *in vivo*, *Was^−/−^* T-cell blasts may still be unable to penetrate into the CNS, as shown by their inability to transfer EAE and by their defective capacity to migrate in response to CXCL12. In fact, it has been previously shown that T cells from *Was^−/−^* mice or from WAS patients display a reduced chemokine-induced chemotaxis due to defective cytoskeletal remodeling [42], [43]. Moreover, WASP has a well-established role in cytoskeletal organization, suggesting that *Was^−/−^* T cells could efficiently adhere in inflamed brain vessels, but fail to spread on the vascular endothelium, a process which is crucial for adhesion strengthening and subsequent lymphocyte transmigration through the endothelial wall [44], [45]. The observation that MOG_35–55_-primed WT T-cell blasts failed to induce EAE in *Was^−/−^* mice could also suggest that WASP is also involved in brain endothelium cytoskeleton remodeling, which is known to be critical for leukocyte transendothelial migration into the tissues [45]. Thus, according with what has been described in other contexts, cytoskeleton modifications required for an efficient entry into the CNS might be altered in WASP-deficiency, and therefore inhibit inflammatory cell transmigration into the CNS parenchyma. Additionally, the inability of MOG_35–55_-primed WT T-cell blasts in transferring EAE in *Was^−/−^* mice could be due to defects in host immune cells, such as CNS resident microglia. In fact, microglial proliferation has been associated with brain lesions in multiple sclerosis [46], [47] and a role of WASP in microglial activation has been reported [48]. In support of these data we also have observed that activated microglia expresses WASP in the CNS of MOG_35–55_-immunized WT mice (data not shown). Lack of WASP could compromise the activation ability of microglial cells and thus contribute to EAE resistance in *Was^−/−^* mice, both in active and adoptive transfer EAE.

Another hypothesis that could explain the resistance against EAE observed in *Was^−/−^* mice is that the absence of WASP may hamper the ability of T and B cells to egress from lymph nodes and reach the target organ, the CNS. Indeed, it has been demonstrated that in *Was^−/−^* mice B cells display a defective migratory ability towards sphingosine 1-phosphate (S1P), a crucial chemoattractant for the positioning of B cells in the splenic marginal zone (MZ) [9]. S1P and its receptors play also an important role in the egress of B and T cells from lymph nodes into the lymphatic circulation (reviewed in [49]). This molecule has recently become an important target in the therapy of CNS autoimmunity, and Fingolimod (FTY720), which upon *in vivo* phosphorylation resembles S1P and impairs lymphocyte trafficking through the receptor subtype S1P1, has shown therapeutic efficacy in EAE [50] and, most importantly, in MS patients [51].

### Conclusions

Although WASP deficiency does not impair the development of myelin-specific autoreactive T cells, it does importantly blunt the ability of these cells to induce disease in the target organ, the CNS. Additionally, the absence of WASP in CNS microglial cells could limit their activation and contribute to EAE resistance in WASP-deficient animals. Understanding the role of WASP in the development of CNS autoimmunity might lead to a deeper comprehension of the molecules and pathways involved in the complex pathology of EAE and MS. Modulation of WASP activation could then become a target for the treatment of CNS inflammatory diseases.

## Supporting Information

Figure S1 **Frequency of WASP-positive T cells in** ***Was^+/−^*** **heterozygous mice.** Frequency of WASP-positive T cells evaluated by flow cytometry in peripheral blood of *Was^+/−^* heterozygous mice before EAE challenge and in spleen and lymph nodes of the same *Was^+/−^* heterozygous mice 14 days after EAE challenge (panel A) and representative histograms showing WASP expression in *Was^−/−^*, *Was^+/−^* and WT mice on peripheral blood samples before EAE challenge (panel B).
(TIFF)
