# Supplementary figures and images for: Development of Central Nervous System Autoimmunity Is Impaired in the Absence of Wiskott-Aldrich Syndrome Protein
Source: PLoS One. 2014 Jan 23;9(1):e86942. doi: 10.1371/journal.pone.0086942 (PMC3900702; doi:10.1371/journal.pone.0086942)

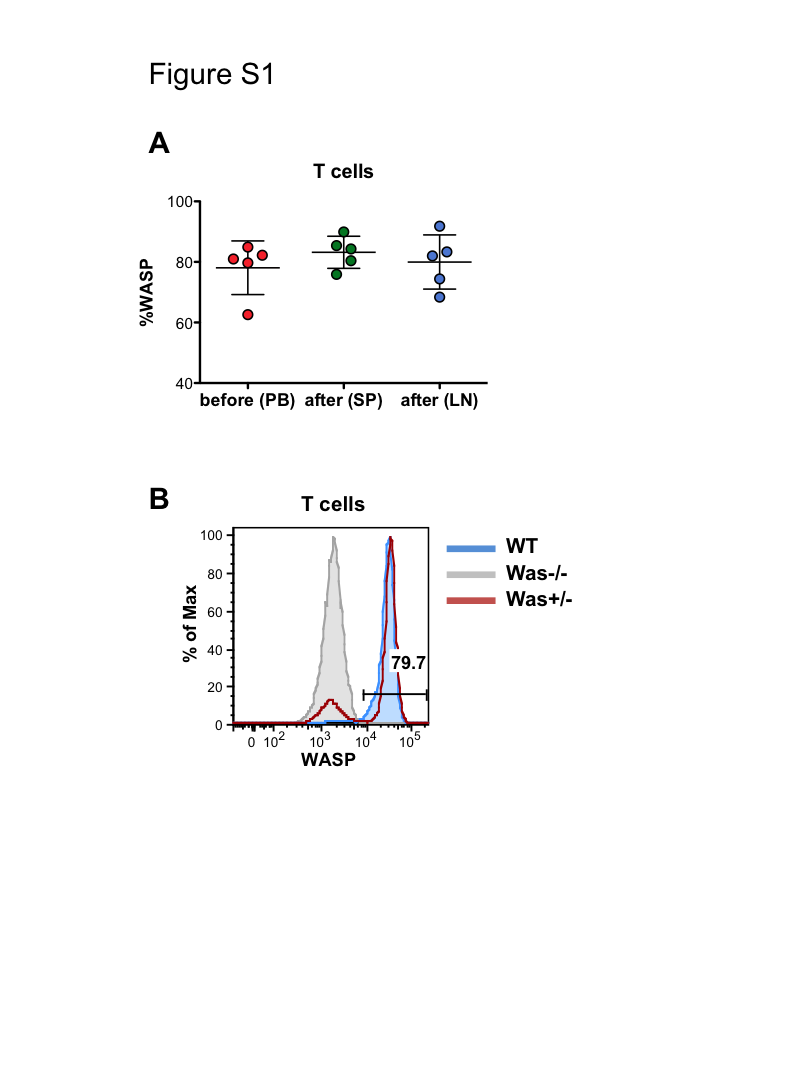

Supplement: Figure S1 — Frequency of WASP-positive T cells in Was+/− heterozygous mice. Frequency of WASP-positive T cells evaluated by flow cytometry in peripheral blood of Was+/− heterozygous mice before EAE challenge and in spleen and lymph nodes of the same Was+/− heterozygous mice 14 days after EAE challenge (panel A) and representative histograms showing WASP expression in Was−/−, Was+/− and WT mice on peripheral blood samples before EAE challenge (panel B). (TIFF) [file pone.0086942.s001.tiff]
